# Wild strawberry shows genetic variation in tolerance but not resistance to a generalist herbivore

**DOI:** 10.1002/ece3.6888

**Published:** 2020-10-10

**Authors:** Minggang Wang, Anne Muola, Peter Anderson, Johan A. Stenberg

**Affiliations:** ^1^ Research Center of Forest Management Engineering of State Forestry and Grassland Administration Beijing Forestry University Beijing China; ^2^ Department of Plant Protection Biology Swedish University of Agricultural Sciences Alnarp Sweden; ^3^ Biodiversity Unit University of Turku Turku Finland

**Keywords:** cotton leafworm, *Fragaria vesca*, jasmonic acid, plant defense syndrome, plant defenses, plant resistance, plant tolerance, resistance breeding, woodland strawberry

## Abstract

Plants’ defenses against herbivores usually include both resistance and tolerance mechanisms. Their deployment has predominantly been studied in either single‐plant genotypes or multiple genotypes exposed to single herbivores. In natural situations, however, most plants are attacked by multiple herbivores. Therefore, aims of this study were to assess and compare the effects of single and multiple herbivores on plant resistance and tolerance traits, and the consequences for overall plant performance. For this, we exposed multiple genotypes of wild woodland strawberry (*Fragaria vesca*) to jasmonic acid (JA), to mimic chewing herbivory and induce the plants’ defense responses, and then introduced the generalist herbivore *Spodoptera littoralis* to feed on them. We found that woodland strawberry consistently showed resistance to *S. littoralis* herbivory, with no significant genetic variation between the genotypes. By contrast, the studied genotypes showed high variation in tolerance, suggesting evolutionary potential in this trait. Prior JA application did not alter these patterns, although it induced an even higher level of resistance in all tested genotypes. The study provides novel information that may be useful for breeders seeking to exploit tolerance and resistance mechanisms to improve strawberry crops’ viability and yields, particularly when multiple herbivores pose significant threats.

## Graphical abstract

Woodland strawberry consistently showed resistance to *Spodoptera littoralis* herbivory, with no significant genetic variation between the genotypes. By contrast, the studied genotypes showed high variation in tolerance, suggesting evolutionary potential in this trait. Prior JA application did not alter these patterns, although it induced an even higher level of resistance in all tested genotypes.

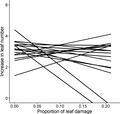

## 1 INTRODUCTION

Plants’ defenses against herbivores can be generally classified as resistance or tolerance. Resistance refers to plants’ ability to avoid or reduce damage caused by enemies (Karban & Baldwin, 1997), while tolerance refers to their capacity to regrow and reproduce following such damage (Strauss & Agrawal, 1999). Resistance and tolerance are sometimes considered interchangeable, as they may provide very similar fitness benefits (Fineblum & Rausher, 1995; van der Meijden et al., 1988). However, individual plants tend to allocate resources to both types of defenses, thereby usually obtaining higher fitness benefits than by allocating the resources to either resistance or tolerance alone (Carmona & Fornoni, 2013; Nunez‐Farfan et al., 2007), indicating that they have complementary effects (Fornoni et al., 2004). The simultaneous expression of both resistance and tolerance traits may be a consequence of fluctuating selection pressures, due to variations in amounts and types of damage (Nunez‐Farfan et al., 2007) and/or the genetic variation in plant defense responses to such damage (Muola et al., 2010). Thus, it is essential to examine both types of defense mechanisms under different damage scenarios, for example, attacks by single and multiple herbivores, and in various plant genotypes to elucidate the variation in plant defenses induced by herbivores.

Plants typically face tremendous variation in damage due to the spatiotemporal variation in the occurrence of herbivores (Tomas et al., 2011). Moreover, different plant genotypes can vary in both tolerance of and resistance to these herbivores, providing opportunities to assess and compare contributions of the two strategies to overall defense trait evolution (Agrawal, 2004; Kant et al., 2008; Muola et al., 2010; Stevens et al., 2007). In addition, the relative importance and roles of resistance and tolerance may differ depending on whether plants are attacked by single or multiple herbivores, as reviewed by Stam et al. (2014). For example, resistance or tolerance may be enhanced or reduced by a previous herbivore, thereby affecting a plant's phenotype, physiology, and induction of its defenses by subsequent herbivores (Omer et al., 2001; Zhu‐Salzman et al., 2008). Such complex induction by multiple herbivores on the overall expression of plant defense can eventually result in diffuse plant performance or fitness as compared to an induction resulting from one herbivore (Ter Horst et al., 2015; Walsh, 2013). Thus, plant resistance or tolerance responses to single and multiple herbivores have been assessed and compared in a few studies, but in most cases, they have been examined in a single genotype (e.g., Dicke et al., 2009; Rodriguez‐Saona et al., 2005; Stam et al., 2014). Few studies have been designed to investigate plants’ defense strategies more comprehensively, by comparing the effects of single and multiple herbivores on the expression of resistance and tolerance traits of multiple plant genotypes (but see Mitchell et al., 2016).

The expression of plant resistance‐ and tolerance‐related traits is regulated by several key signaling pathways involving phytohormones, including jasmonic acid (JA) (Glazebrook, 2005), salicylic acid (Zarate et al., 2007), and ethylene (Stotz et al., 2000). Among these phytohormones, JA particularly has long recognized roles in inducing plant defenses against chewing herbivores, for example, by triggering enhanced production of plant secondary metabolites that deter or reduce further herbivore feeding (Thaler et al., 1996). Exogenous applications of JA have been shown to induce resistance to insect herbivores in a wide range of plant species **(**e.g., Omer et al., 2000; Délano‐Frier et al., 2004; Zhu & Tian, 2012). For example, in tomato it can induce increases in levels of proteinase inhibitors and polyphenol oxidase, resulting in reductions in performance of many pest insects in the field (Thaler et al., 1996). JA also has regulatory effects on plant development and physiology (Creelman & Mullet, 1995; Santino et al., 2013), which may directly alter the expression of either tolerance or resistance traits, or change the inducibility of plant defenses by later herbivore attacks. Hence, JA may influence plants’ productivity and reproduction (Avanci et al., 2010; Délano‐Frier et al., 2004), particularly when plants are subjected to subsequent herbivore feeding.

In the study presented here, we exposed multiple woodland strawberry (*Fragaria vesca* L.) genotypes to larvae of the polyphagous moth *Spodoptera littoralis* to detect the genetic variation in plant resistance to, and tolerance of, a generalist chewing herbivore, and to examine whether these defenses and the potential genetic variation in them is modified by prior application of JA. We posed three specific hypotheses: first, that the resistance and tolerance of *F. vesca* to *S. littoralis* would vary among the plant genotypes; second, that the resistance and tolerance responses to *S. littoralis* would be modified (genotype‐dependently) by prior application of JA; and third, that the plants’ performance would be promoted by prior application of JA, either through direct developmental or physiological effects, or indirectly through inducing enhanced resistance to, and tolerance of, *S. littoralis*.

## 2 MATERIALS AND METHODS

### 2.1 Study system

Woodland strawberry, *F. vesca*, is a herbaceous perennial species that is widely distributed in the northern Hemisphere (Hilmarsson et al., 2017). It is a wild relative of cultivated garden strawberry (*Fragaria × ananassa* Duch.) that has been used as a model system in many studies concerning pest management or evolutionary consequences of domestication (e.g., Badmi et al., 2019; Egan et al., 2018; Muola et al., 2017; Osorio et al., 2008).

The cotton leafworm, *S. littoralis* (Lepidoptera: Noctuidae), is a polyphagous moth originating from Africa that is known to induce and respond to plant resistance (Anderson et al., 2001, 2011). It has a broad range of host plants and is known to attack more than 130 plant species from 56 families, including important crops such as cotton and tomato (Pogue, 2002). *Spodoptera* spp. have been recorded as strawberry pests worldwide, including Europe (El‐Sheikh, 2015), America (Montezano et al., 2018), and Asia (Yang et al., 2016). Although *S. littoralis* has not yet been found in Sweden, this species is a range‐expanding pest in Europe that is characterized by extreme polyphagy and strong adaptive ability during host plant selection (Proffit et al., 2015). The purpose of this study was to examine the genetic variation in plant defense responses to generalist chewing herbivores, represented by *S. littoralis*. For this purpose, *S. littoralis* eggs were hatched and the larvae were reared on an artificial diet (Hinks & Byers, 1976), in a growth chamber providing 16:8‐hr light: dark cycles, at 25°C and 70% RH, until the 3rd instar before introduction to the experimental plants. The site of these, and all the procedures described below, was the Swedish University of Agriculture's campus at Alnarp.

### 2.2 Experimental design

To assess genetic variation in woodland strawberry's resistance to, and tolerance of, feeding damage by the generalist herbivore *S. littoralis*, and possible modulation of its responses by prior application of JA, we subjected 16 genotypes of the species (described by Weber et al., 2020) to four treatments (see below). The genotypes were selected from our archive of clones collected from various sites in Uppland, Sweden, in 2012 (Weber et al., 2020). Plant material for the experiment was propagated from runners collected from the clone archive that were maintained in a greenhouse providing 16‐hr light: 8‐hr dark, 20:16°C cycles. On 5 May, 2018, at least 28 replicates per genotype were cloned for this experiment. Runners were planted in pots (7 × 7×7 cm) with 0.3 L Hasselfors™ potting medium (Hasselfors Garden, Örebro, Sweden). Twelve weeks later, the young plants were assigned to one of four treatments following a full‐factorial design, described below and designated: (a) control, (b) JA, (c) *S. littoralis*, and (d) JA + *S. littoralis*. Seven replicates of each genotype were subjected to each treatment, resulting in a total of 448 (4 × 16×7) pots. The pots were placed in a greenhouse (providing 16‐hr light: 8‐hr dark, 20:16°C cycles) in a randomized block design to minimize confounding effects of potential environmental gradients (e.g., in temperature, evaporation, and light intensity). Each block included one replicate of each genotype subjected to each of the four treatments.

Before the application of JA, all plants were covered by 2‐L plastic bags. Each plant assigned to the JA or JA+ *S. littoralis* treatments was treated with 1 mM JA solution, made by dissolving 5 ml of a 210 g JA/mol EtOH solution in 2,365 ml of Milli‐Q water and then adding 2,378 ml of Tween 20. Plants assigned to the control and *S. littoralis* treatments were sprayed with control solution containing the same amounts of Milli‐Q water and Tween 20 supplemented solely with 5 ml pure EtOH. JA and control solutions were carefully sprayed in the covering bags to avoid cross‐pot contamination of JA. The spray was applied three times, using a sprayer from the top of the plant to ensure that all leaves were saturated. Each individual plant received roughly 0.8 ml of JA solution (containing 2.4 µmoles of JA, Sigma) or control solution. The bags were closed immediately after the application of solutions for 48 hr, to allow the solutions to settle on the plant surfaces.

Four days after the treatments, all plants were individually covered with perforated bags (supplied by Baumann Saatzuchtbedarf). One 3rd instar *S. littoralis* larva (25.6 ± 3.2 g) was introduced into each bag covering a plant assigned to the *S. littoralis* or JA+ *S. littoralis* treatments. Larvae were starved for 24 hr in an Eppendorf tube before weighed and then released next to the basal part of a bagged plant by opening the cap of the Eppendorf tube. Two hours after introduction of the larva, we checked all the tubes. Each larva could move freely in the bag it was placed in, but if it caused no damage, it was replaced by another one. Plants were carefully watered daily at their bases, to avoid affecting the larvae.

The larvae were allowed to feed on the experimental plants for a week before being removed together with the perforated bags. Mortality rates of the larvae were very high (101 and 107 dead out of 112 individuals for – JA and + JA treatment, respectively), so instead of estimating their growth rates, we recorded the larval mortality and the proportion of the area of every leaf of each plant damaged by *S. littoralis* feeding. The consumed area was visually estimated and averaged as the overall damage proportion per plant. The estimation was also used to assess the plant genotypes’ tolerance of *S. littoralis* damage given the relatively high variation in such damage among genotypes (3.1%–12.5% and 2.6%–7.0% under –JA and +JA treatment, respectively). The inverse of the proportion of damaged leaf area of a plant was used as a proxy for its resistance to *S. littoralis*. To assess the plants’ tolerance of the *S. littoralis* damage, they were allowed to grow in the same greenhouse for four additional weeks. After that, each plant's leaves were counted again to measure its regrowth (increase in leaf number) during a four‐week recovery period. The regrowth after damage was used as an estimate of plant tolerance of *S. littoralis*. Then, all shoot tissues were cut at each plant's base, at soil surface level, and the harvested shoots were immediately oven‐dried at 70°C for 4 days, and weighed to determine its aboveground biomass.

### 2.3 Statistical analyses

Our experiment was designed with random factors (genotypes and blocks), so we used mixed models to test for significant random effects (Littell, 2002). We were particularly interested in genetic variation in plant resistance and tolerance, as well as plant performance following JA application and/or insect feeding. A general linear mixed model (GLMM) with a maximum‐likelihood (ML) iterative algorithm was used to analyze the plant resistance and tolerance data. Plant resistance was estimated as the inverse of leaf area damaged by *S. littoralis*. In the analysis, the proportion of damaged leaf area of plants exposed to *S. littoralis* herbivory was included as a response variable and presence/absence of JA application as an explanatory fixed factor. Plant genotype and its interactions with JA, as well as block, were included as random factors. Initial plant size (leaf number after larva damage) and the mortality of larvae (0/1) were included as covariates. Plant tolerance was expressed as the slope of the reaction norm relating a plant's regrowth (increase in leaf number) after *S. littoralis* damage to the plant's proportion of damaged leaf area. Similarly, in this analysis, only plants that were assigned to *S. littoralis* were included. The increase in leaf number after damage was used as response variable, and we included JA application (±) as a fixed factor; plant genotype and its interaction with JA, as well as block, as random factors; and the proportion of damaged leaf area, mortality of larvae (0/1), and initial plant size as covariates. A significant effect of plant genotype, according to this model, would indicate genetic variation in plant resistance and/or tolerance, and a significant interaction between genotype and JA would indicate significant differences in the tested plant genotypes responses to the JA treatment.

Another GLMM was used to estimate the effects of JA application and insect feeding on plant performance. In this model, we used shoot biomass at the final harvest as an estimate of plant performance. The shoot biomass of plants that were exposed to all four treatments (full‐factorial cross of ±*S. littoralis* feeding and ±JA application) was included as response variable. Insect feeding and JA application (±) were included as fixed factors, while plant genotype and its interactions with fixed factors (as well as block) were treated as random factors. Initial plant size was used as a covariate. The significance of the random factors was tested with the likelihood‐ratio chi‐square test (Zuur et al., 2009). Additionally, to examine the relationship between plant performance and plant resistance/tolerance across genotypes, we again used GLMMs, in which only plants exposed to insect feeding (*+S. littoralis*) were included for analyses. In the models, shoot biomass of these plants was used as the response variable, with the proportion of leaf area (for resistance) and increase in leaf number after damage (for tolerance) used as predictors. In both models, plant genotype and its interaction with the predictor were included as random factors. To meet the assumptions of normality and homogeneity of data residuals for the GLMMs, data on proportion of leaf area damaged and plant biomass were square‐root transformed. All the analyses were carried out using the *lmer* function in the “lme4” package in R version 3.4.1. (R Core Team 2016).

## 3 RESULTS

### 3.1 Proportion of leaf area damaged by *S. littoralis* following JA application

**Figure 1:**
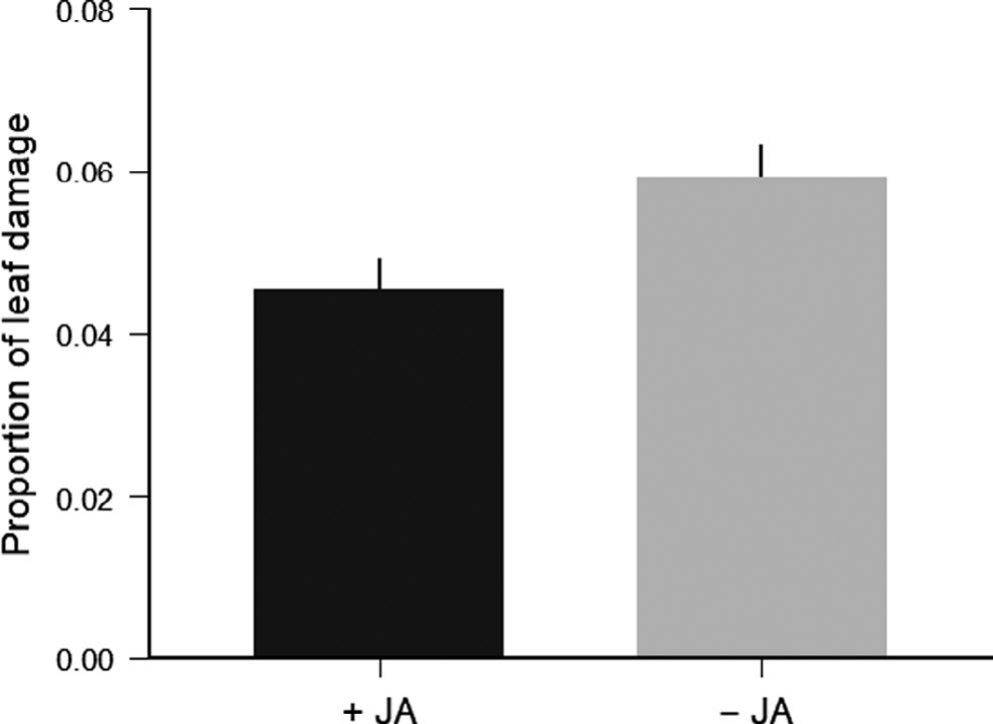
Proportion of leaf area (mean ± *SE*) damaged by *Spodoptera littoralis* of *Fragaria vesca* genotypes that were treated with jasmonic acid (+JA, black bar) or served as controls (‐JA, gray bar). The inverse of the proportion of damage was used to estimate the resistance of plant genotypes. The difference in proportion of damage between the JA‐treated plants and controls was significant at the *p* < .05 level

The proportion of leaf area damaged by *S. littoralis* did not significantly differ among genotypes (*genotype*: *X* ^2^ = 1.49, *p* = .222, Figure S1), suggesting that there was no genetic variation in plant resistance to *S. littoralis*. Likewise, the effect of prior application of JA did not differ among genotypes (*JA* × *genotype*: *X* ^2^ = 0.00, *p* = 1.00, Figure S1). However, the proportion of damaged leaf area was reduced by 40% by the prior application of JA (mean ± *SE*: −JA 6% ± 0. 6% vs. +JA 4.5% ± 0. 4%; *F*_1, 198_ = 5.38 *p* = .021, Figure 1) and strongly dependent on whether the *S. littoralis* survived through the feeding trial (*F*_1, 218_ = 26.7, *p* < .001). Smaller plants suffered proportionally more damage than the larger plants (*F*_1, 214_ = 5.08, *p* = .025).

### 3.2 Plant tolerance to *S. littoralis* damage following JA application

**Figure 2:**
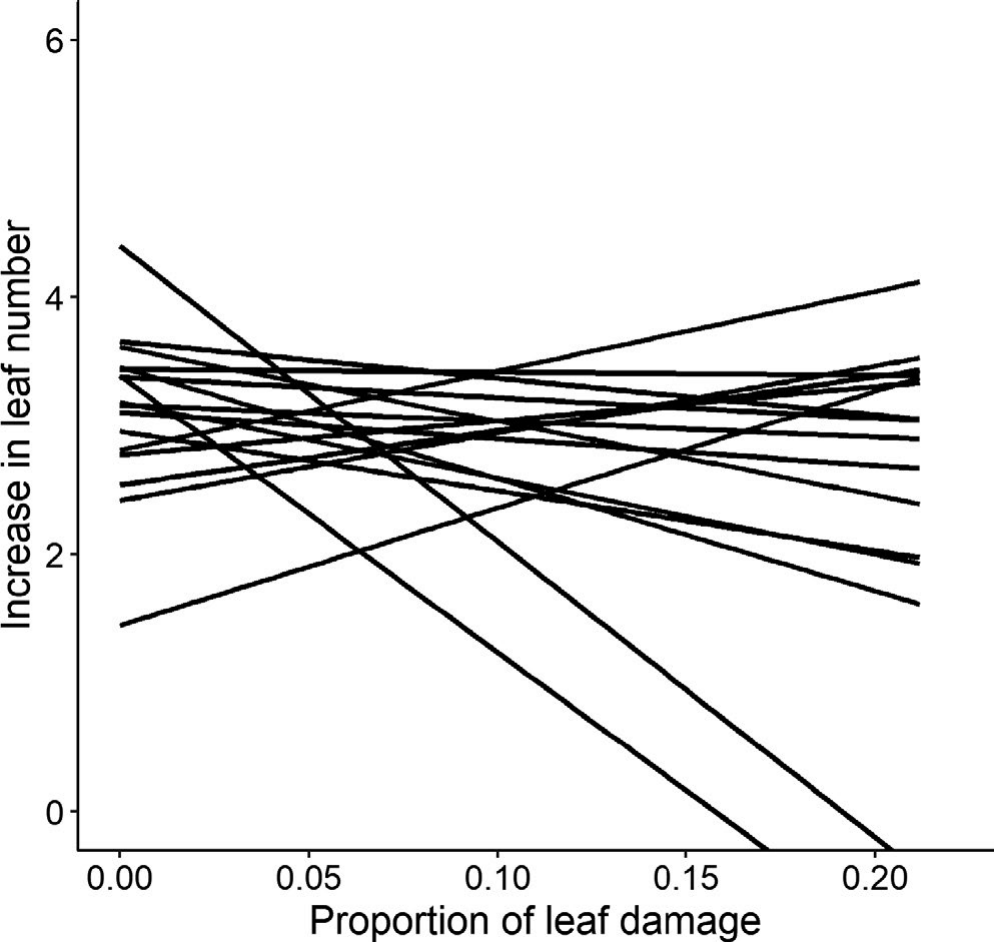
Genetic variation in tolerance of *Fragaria vesca* genotypes to damage by the insect herbivore *Spodotera littoralis*, estimated from mean increases in leaf number relative to the mean proportion of damaged leaf area (*n* = 7), during regrowth for four weeks after the damage. Each line represents results obtained for one plant genotype. There were significant differences in this respect among genotypes at the *p* < .05 level

Each plant's tolerance was expressed as the slope of the reaction norm between its regrowth (increase in leaf number) after damage and its level of herbivore damage. JA application did not influence the overall tolerance of the plant genotypes to *S. littoralis* (*F*_1, 198_ = 0.70, *p* = .403), neither did the initial plant size at the introduction of *S. littoralis* impact the overall tolerance (*F*_1, 219_ = 0.03, *p* = .862). However, we found significant variation in plant tolerance of damage by *S. littoralis* among genotypes (*X^2^* = 6.32, *p* = .012, Figure 2). Genetic variation in tolerance was not dependent on the application of JA (*JA* × *genotype*: *X^2^* = 0.00, *p* = 1.00) nor on the damaged leaf area by *S. littoralis* (*F*_1, 213_ = 0.21, *p* = .650).

### 3.3 Plant performance following JA application and *S. littoralis* damage

**Figure 3:**
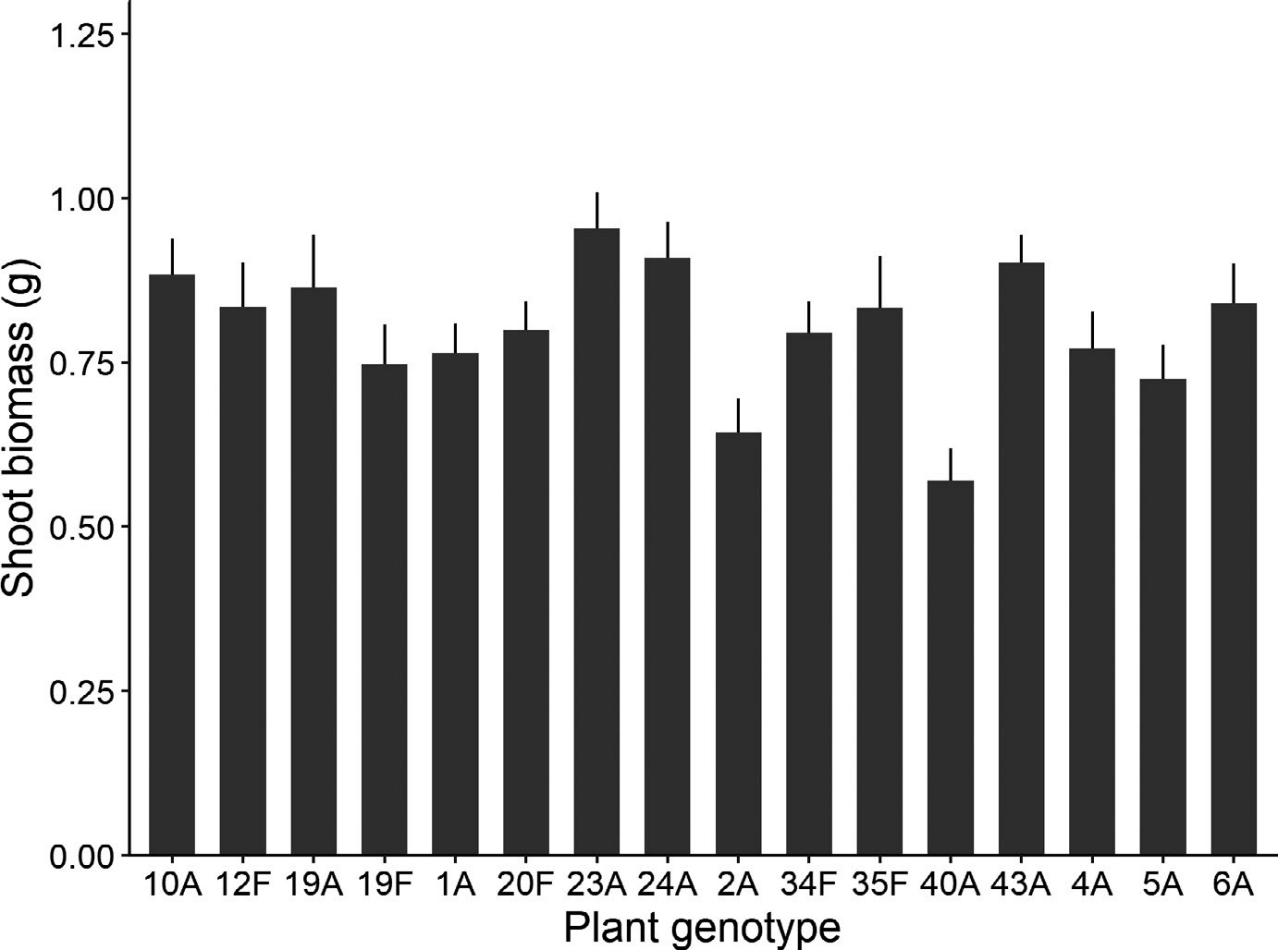
Shoot biomass (mean ± *SE*) of 16 *Fragaria vesca* genotypes, regardless of the presence or absence of JA or insect feeding. Plant shoot biomass was used to estimate the plant performance of each genotype under insect feeding. There were significant differences in this respect among genotypes at the *p* < .01 level

Plant shoot biomass at harvest was used as a measure of plant performance following JA and insect‐feeding treatments. Overall, JA application did not influence the final shoot biomass of plants across genotypes (*F*_1, 425_ = 0.08, *p* = .775), but feeding by *S. littoralis* marginally reduced the shoot biomass by 4.6% of all the genotypes (*F*_1, 425_ = 4.38, *p* = .053, Figure S2). We found significant genetic variation in plant performance (*X* ^2^ = 7.94, *p* = .005, Figure 3), regardless of the prior application of JA or insect feeding (*JA* × *genotype*, *X* ^2^ = 0.00, *p* = 1.00; *insect feeding* × *genotype*, *X* ^2^ = 0.29, *p* = .591). The initial size of plants prior to treatments significantly affected plant shoot biomass, with larger plants having higher biomass at the end of experiment, irrespective of their genotype (*F*_1, 444_ = 58.03, *p* < .001). The shoot biomass of a genotype was neither related to the damaged leaf area it experienced nor to its regrowth after the damage (both leaf damage/regrowth × *genotype*: *X* ^2^ = 0.00, *p* = 1.00).

## 4 DISCUSSION

Exposing F. vesca to damage by S. littoralis resulted in differences among the genotypes in regrowth after damage, but not in the proportion of damaged leaf area. These results indicate genetic variation in tolerance of F. vesca, but not in its resistance, to this generalist herbivore. However, *F. vesca* is known to be able to tolerate folivory/leaf damage by other herbivores as well (Muola & Stenberg, 2018), and thus, it remains to be tested whether the observed genetic variation in tolerance was a specific response to *S. littoralis* damage or to the leaf removal in general. Furthermore, not only the herbivore species but also the amount of damage can affect plant defense responses and the genetic variation in them (Karban & Baldwin, 1997). To verify whether the observed genetic variation in plant tolerance is independent of the amount of damage, further studies are needed in which plant tolerance responses are examined when a gradient of proportion of leaf area is experimentally removed from these genotypes (Fornoni & Nunez‐Farfan, 2000). We also detected genetic variation in plant performance, although the performance of a genotype was not strongly correlated with its tolerance to the generalist herbivore damage. The lack of association between plant tolerance and performance at the genotype level suggests that the tolerance mechanisms of *F. vesca* may not be sufficient for the species to counter herbivore damage (as applied in this study) and maintain fitness. Another simple possible explanation is that regrowth after damage may not be solely responsible for the observed tolerance response, and other mechanisms may also be involved. For instance, in a previous study with woodland strawberry and a leaf chewing herbivore, it was observed that plants could increase their photosynthesis rates after they were damaged (Muola & Stenberg, 2018). Regardless of these possibilities, the genetic variation in tolerance suggests that tolerance traits have evolutionary potential in wild strawberry populations.

Plant resistance has received more attention than plant tolerance in studies of plant defenses against insect herbivores (Karban, 2011). Many plant species show genetic variation in their resistance responses to herbivores (e.g., Bossdorf et al., 2005; Muola et al., 2010; Weber et al., 2020). In particular, Weber et al. (2020) found high variation among the plant genotypes used in this study in resistance to a more specialized and coevolved herbivore (*Galerucella tenella* L.). The generally high mortality of *S. littoralis* feeding on all the plant genotypes observed in this study may reflect this herbivore's polyphagy, and lack of evolutionary history with woodland strawberry. The lack of significant genetic variation could potentially be explained by the overall high variation in damaged leaf area within genotypes (Figure S1) and relatively low replication (*n* = 7 per genotype). The overall high variation in damaged leaf area within genotypes (Figure S1), which was strongly related to initial plant size and the mortality of *S. littoralis* larvae, may also have contributed to the lack of detected genetic variation. Accordingly, larger plants tend to receive less proportional damage from herbivores, or have stronger physical or physiological vitality to resist them, than small plants (Vandegehuchte et al., 2010). The high mortality of *S. littoralis* larvae likely contributed to the high variation in leaf damage through, for instance, differences among individuals of the same genotype in the duration of larval feeding. However, to fully understand the reasons for the apparent absence of genetic variation in resistance, further studies are needed in which the size of plants is standardized and the number of replicated plants is increased within each genotype before exposure to *S. littoralis* herbivory. Furthermore, it is noted that plants in our study were relatively young, which may be another explanation for the presence of tolerance but absence of resistance given the ontogenetic dependence of many plant species on their defense responses (Muola et al., 2010; Visschers et al., 2019; Wang et al., 2018).

Numerous studies indicate that exogenous application of JA on plant leaves, roots, or seeds can increase plants’ resistance to insect herbivory (Paudel et al., 2014; Thaler et al., 2001; War & Sharma, 2014; Zhang et al., 2011). Similarly, we found that exogenous application of JA on *F. vesca* leaves reduced the overall proportion of leaf area consumed by *S. littoralis*, suggesting successful induction of plant resistance, but the levels of resistance induced by JA application were similar among all genotypes. A possible explanation for the lack of genetic variation in the resistance of *F. vesca* induced by exogenous JA is that it involves a strongly conserved defense mechanism from which natural selection has removed genetic variation. However, although JA is usually regarded as an effective plant defense inducer, it does not have identical effects to actual insect herbivory, due to the lack of salivary elicitors of defenses and specific elements of interactions between herbivores and host plants (Hogenhout & Bos, 2011; Louis et al., 2013). Thus, the generality of JA’s effects may have contributed to the lack of detected genetic variation of *F. vesca* in the resistance and tolerance responses it induced.

Overall, the performance of *F. vesca* plants tended to be reduced by the feeding of *S. littoralis,* indicating that generalist insect species may impair their fitness and impose associated selection pressures. However, although we detected genetic variation in plant performance, it was not affected by either application of JA or insect feeding. Clearly, neither the induction of resistance by JA nor the genetic variation in plant tolerance to *S. littoralis* feeding had significant effects on the overall performance of *F. vesca* in our study. Taken together, our results indicate that *F. vesca* shows genetic variation in tolerance of, rather than resistance to, attack by this generalist herbivore. Thus, the results provide little support for our second and third hypotheses, but partial support for our first hypothesis. As *F. vesca* is a wild relative of garden strawberry (*Fragaria × ananassa*), this study may provide useful information for breeders seeking to exploit tolerance and resistance mechanisms to improve strawberry crops’ viability and yields, particularly when multiple herbivores pose significant threats.

## CONFLICT OF INTEREST

The authors declare that they have no conflicts of interest.

## AUTHOR CONTRIBUTION

**Minggang Wang:** Conceptualization (lead); Data curation (lead); Formal analysis (lead); Investigation (lead); Methodology (lead); Project administration (equal); Writing‐original draft (equal); Writing‐review & editing (equal). **Anne Muola:** Conceptualization (equal); Funding acquisition (lead); Project administration (equal); Writing‐original draft (equal); Writing‐review & editing (equal). **Peter Anderson:** Conceptualization (equal); Methodology (equal); Writing‐review & editing (equal). **Johan A Stenberg:** Conceptualization (equal); Funding acquisition (lead); Project administration (lead); Writing‐original draft (equal); Writing‐review & editing (equal).

## Supporting information

FigS1‐S2

## Data Availability

Data collected in the research are available at Dryad https://doi.org/10.5061/dryad.qjq2bvqdf
