# Supplementary material for: Wild strawberry shows genetic variation in tolerance but not resistance to a generalist herbivore
Source: Ecol Evol. 2020 Oct 10;10(23):13022–9. doi: 10.1002/ece3.6888 (PMC7713946; doi:10.1002/ece3.6888)

**Wild strawberry shows genetic variation in tolerance but not resistance to a generalist herbivore**

Minggang Wang^1, 2*^, Anne Muola^2,3^, Peter Anderson^2^, Johan A. Stenberg^2^

^1^Research Center of Forest Management Engineering of State Forestry and Grassland Administration, Beijing Forestry University, Beijing, China

^2^Department of Plant Protection Biology, Swedish University of Agricultural Sciences, Alnarp, Sweden

^3^Biodiversity Unit, University of Turku, Turku, Finland

Corresponding author:

**Dr. Minggang Wang**

Research Center of Forest Management Engineering of State Forestry and Grassland Administration, Beijing Forestry University, Beijing, China

Email: minggang.wang@slu.se

**Supporting information**

**Figure S1.** Proportion of leaf area (mean ± SE) damaged by *Spodoptera littoralis* of 16 *Fragaria vesca* genotypes that were treated with jasnomic acid (+ JA, grey bars) or served as controls (- JA, black bars).


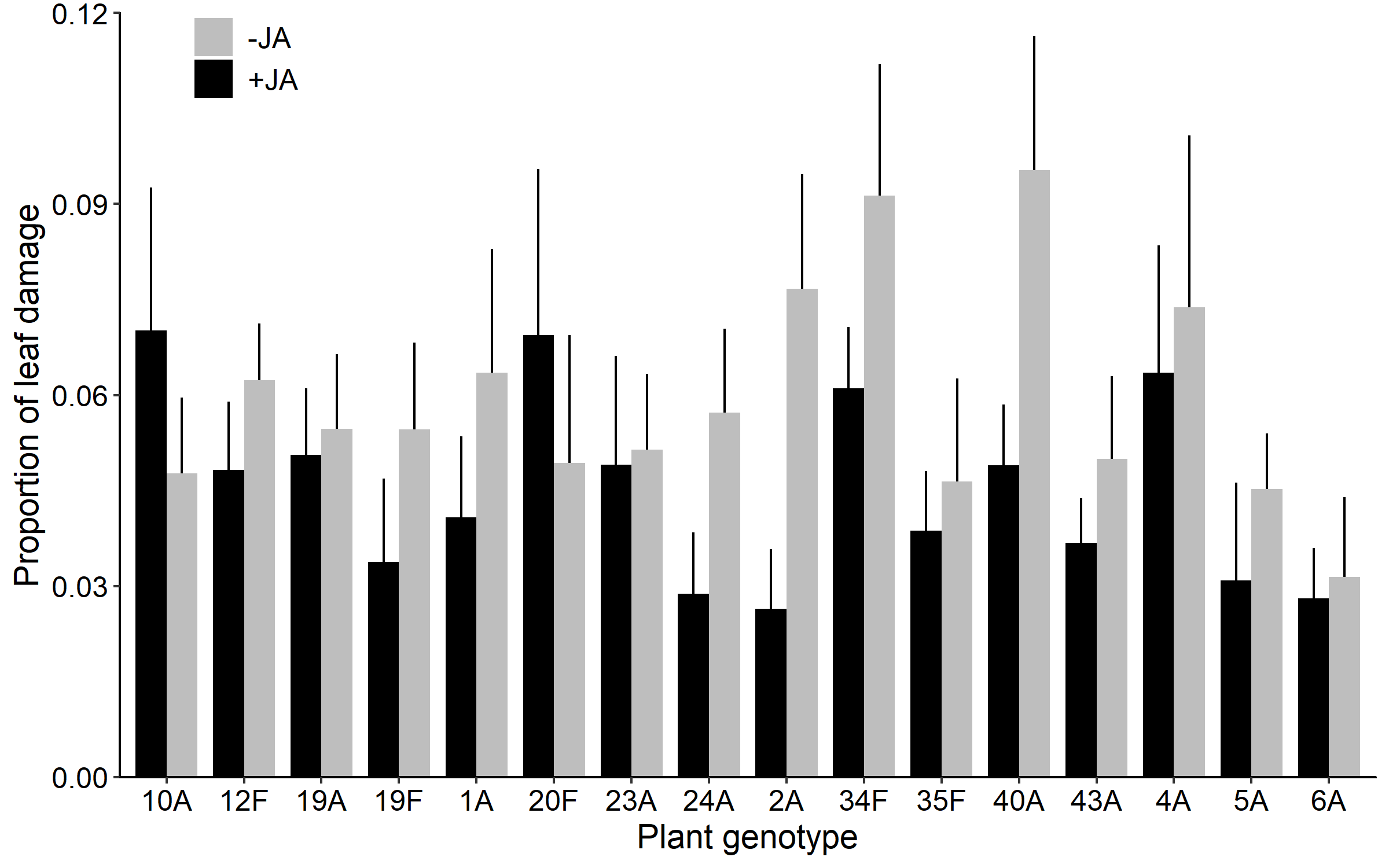


**Fig. S2** Shoot biomass (mean ± SE) of *Fragaria vesca* genotypes that were previously damaged by the herbivore *Spodotera littoralis* (+ Insect feeding) or served as controls (- Insect feeding). Plant shoot biomass was used to estimate the performance of each genotype under insect feeding.


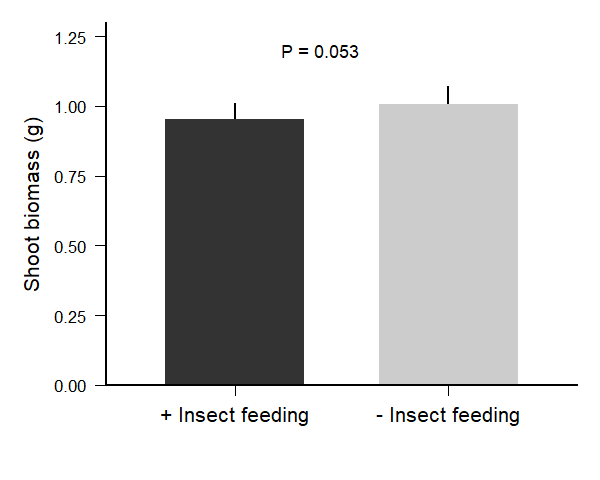

Supplement: Supplementary file 1 — FigS1‐S2 [file ECE3-10-13022-s001.docx]
